# A bedside technique and historical aspects of the cutaneous findings in scurvy

**DOI:** 10.1016/j.ijscr.2020.04.105

**Published:** 2020-05-21

**Authors:** Steven H. Yale, Halil Tekiner, Joseph J. Mazza, Eileen S. Yale

**Affiliations:** University of Central Florida College of Medicine, 6850 Lake Nona Blvd, Orlando, FL 32827, United States; Department of the History of Medicine and Ethics, Erciyes University School of Medicine, Talas, Kayseri 38280, Turkey; Marshfield Clinic Research Foundation, Marshfield, WI 54449, United States; University of Florida, Division of General Internal Medicine, 2000 SW Archer Rd., Gainesville, FL 32608, United States

To the Editor:

We commend the authors for reporting a child initially believed to have osteomyelitis, but later diagnosed as subperiosteal bleeding secondary to scurvy, a disease due to ascorbic acid (vitamin C) deficiency [1]. This case serves as an important reminder that disease present in bones may reflect a systemic process. In addition to its effects on bone metabolism, ascorbic acid is important to the structure of collagen, the major component of connective tissue. Procollagen secreted by fibroblasts provide the tensile strength required by numerous tissues to maintain vascular integrity, wound integrity and healing, muscle strength, and dental and mucous membranes. Deficiency leads to spontaneous bleeding, weakness, loss of dentition, hair and bone manifestation. The skin and mucous membranes are always involved and should be systematically evaluated. Scurvy although once considered an obsolete disease still exists. Thus, it is incumbent that physicians recognize key cutaneous physical findings, particularly those considered pathognomonic for the disease. Herein, we remind physicians about a simple bedside technique to further assist in diagnosis and skin findings based on a historical approach.

Carl Leede (1882–1964), an assistant at the Hamburg-Eppendorf Hospital, Germany, under the aegis of senior physician Dr. Theodor Rumpel (1862–1923), recognized the difficulty confirming scarlet fever in the absence of the exanthema. He described in 1911 the “stasis test” as a method of diagnosing scarlet fever [2]:

In each case I determined the systolic and diastolic blood pressure and then filled the cuff to a pressure considerably below the diastolic pressure (usually 45–60 mm of mercury). I leave the cuff with the tube disconnected and examined the area within 5 minutes for bleeding, which typically occurs in 5–20 minutes. (…) In the cases of other diseases which I have examined, only exceptionally has similar bleeding been observed by this method. Only in cases of measles did I obtain values which approached those found in scarlet fever (p. 294). (…) I had the impression as if the capillaries in measles are not quite as involved as in scarlet fever. I believe I have demonstrated that in scarlet fever, almost without exception, there is a pathologically increased vulnerability of the capillaries and I believe that this symptom can be used diagnostically. In my experience, a negative stasis test can be used as an almost sure criterion against scarlet fever while a positive test should only be used together with the other symptoms (p. 295).

In 1914, Alfred Fabian Hess (1875–1933) with the assistance of Miss Mildred Fish, described a method to assess capillary fragility referred to as the “Hess Test” or capillary resistance test in the diagnosis of scurvy in infants [3]:

[s]ubjecting the capillaries and vessels of the arm to increased intravascular pressure, by means of an ordinary blood-pressure band, and of observing whether this strain results in the escape of blood through the vessels—the appearance of petechial hemorrhages into the skin. The vessels of normal infants were found to withstand, without apparent disturbance, 90 degrees of pressure for three minutes, whereas the vessels of infants suffering from scurvy gave way under this pressure. The test is not specific for scurvy, but is a method of demonstrating a weakness of the vessel walls, whatsoever may be its cause (p. 405).

Leede thus reported this finding in patients with scarlet fever not scurvy. In the literature this test is erroneously referred to as the Rumpel-Leede test rather than Hess and Fish test, as they were the first to have elicited petechial hemorrhages in cases of scurvy.

Ludwig Aschoff (1866–1942) and Walter Karl Koch (1880–1962) in 1919 described the “corkscrew” appearance of the hair in patients with scurvy [4]:

Hair follicles in this disease are associated with curvy hair. The hair breaks off easily, folds like a corkscrew, appears to be flattened, sinks into the follicle, or is completely absent. With a magnifying glass you can see in the middle of the gray-white tips of the hair follicles mentioned, the remainder of a clear hair shaft, which seems to be broken and rolled up in a ring-like shape in the follicular substance. If you run your fingertips gently over the altered skin, you clearly feel a blunt grater (p. 15).

Harold Waterlow Wiltshire (1879–1937) in his evaluation of over 3000 troupes in the Serbian army in 1919 recognized follicular hyperkeratosis to be an early finding in patients with scurvy [5]:

The hyperkeratosis was found in the vast majority of cases of clinical scurvy and was not found in other conditions except among men living on the same dietaries as those in whom scurvy developed, that is, on diets proved to be deficient in the antiscorbutic vitamin. Since the condition develops some weeks before clinical scurvy appears should prove of great diagnostic value and should aid in the prevention of clinical scurvy before many cases had developed among bodies of men (p. 565)·

We believe that it was Roslyn B. Alfin-Slater (1916–2002) and David Kritchevsky (1920–2006) in 1980 that coined the term “swan-neck” deformity to describe the other, less common, appearance of the hair follicle in scurvy [6].

The origin of these clinical signs provides an appreciation for those that identified and described these key cutaneous findings, providing a window into the recognition of this disease.

## Declaration of Competing Interest

Drs. Steven H. Yale, Halil Tekiner, Joseph J Mazza and Eileen S. Yale report no conflict of interest.

## Funding

No source of funding.

## Ethical approval

The article is a letter to the editor and does not require ethical approval.

## Consent

Not Applicable.

## Author contribution

Drs. Steven Yale, Halil Tekiner, Joseph J Mazza and Eileen S. Yale were involved in writing the paper.

## Registration of research studies

1. Name of the registry: Not Applicable.
2. Unique identifying number or registration ID: Not Applicable.
3. Hyperlink to your specific registration (must be publicly accessible and will be checked): Not Applicable.

## Guarantor

Steven H. Yale MD.

Halil Tekiner PhD.

Joseph J Mazza MD.

Eileen S. Yale MD.
